# Pleomorphic carcinoma of the breast: A case report and review of the literature

**DOI:** 10.1016/j.ijscr.2025.111486

**Published:** 2025-06-07

**Authors:** Nobuyuki Takemoto, Masanori Yasuda, Kousuke Shimanaka, Hiroshi Yamamoto

**Affiliations:** a Department of Breast & Thyroid Surgery, Japan Medical Alliance East Saitama General Hospital, 5-517, Yoshino, Satte-City, Saitama-Pref, Japan; b Department of Pathology, Japanese Red Cross Gifu Hospital, 36-3, Iwakuramachi, Gifu-City, Gifu-Pref, Japan

**Keywords:** Pleomorphic carcinoma, Breast, Rapidly growing, Differential diagnosis

## Abstract

**Introduction and importance:**

Pleomorphic carcinoma of the breast is extremely rare, accounting for <0.1 % of breast malignancies. It has a poor prognosis, but its clinicopathologic features are not well characterized.

**Case presentation:**

A 49-year-old woman was diagnosed with bilateral breast cancer (rt: cT2cN0M0 cstageIIA, lt: cT2cN1M0 cstageIIB) and its intrinsic type was non-luminal type (right breast) and lumina A-like (left breast). After neoadjuvant chemotherapy, the patient achieved pathologic partial response and underwent breast-conserving surgery with axillary lymph node dissection and radiotherapy. After 6 years and 7 months, local recurrence in the right breast was observed, and mastectomy was performed. However, after 4 weeks, metastasis to the contralateral breast was found, and a further 13 weeks later, 70 % of the thoracic cavity was occupied by metastatic pleural tumors. Pathological findings suggested pleomorphic carcinoma. The patient passed away 25 weeks later after the diagnosis of local recurrence.

**Clinical discussion:**

Pleomorphic carcinoma could represent an extreme end of dedifferentiation of invasive breast carcinoma of no special type or part of the differentiation of metaplastic spindle cell carcinoma. It is also important to differentiate this from malignant tumors such as sarcomas with giant cells or spindle cells, and metastatic tumors. Pathological diagnosis requires lack of heterologous non-epithelial components, and strong immunostaining with epithelial markers.

**Conclusion:**

When a rapidly growing breast mass is encountered, pleomorphic carcinoma should be considered as one of diagnostic candidate. More case accumulation and analyses are awaited to further characterize this carcinoma.

## Highlights

• Pleomorphic carcinoma (PC) accounts for <0.1 % of all breast malignancies.
• It is important to differentiate PC from malignant tumors (e.g., sarcoma).
• When a rapidly growing breast mass is encountered, PC should be considered.

## 1 Introduction

The term “invasive breast carcinoma (IBC) of no special type (NST)” refers to a large and heterogeneous group of IBCs that cannot be classified morphologically as any of the special histological types [1]. Pleomorphic carcinoma (PC) belongs to this group, and is a rare pattern of high-grade IBC-NST characterized by proliferation of pleomorphic and bizarre tumor giant cells constituting >50 % of the tumor cells [1], with only three previous reports examining >10 cases [[2], [3], [4]]. The clinical course of PC is rapid and aggressive, with a poor prognosis [[2], [3], [4]]. However, because there are only a few reported cases, little research has been done, especially on imaging and treatment methods. We therefore report a case of breast cancer in which invasive ductal carcinoma transformed into highly malignant PC, resulting in rapid progression and poor prognosis. This manuscript has been reported in line with the SCARE criteria [5].

## 2 Case

**Fig. 1:**
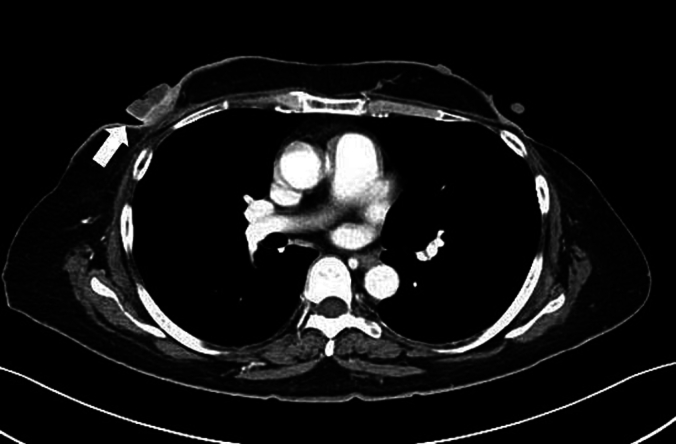
Enhanced CT; the right nipple is swollen and has a mass-like appearance compared to the opposite side. A slightly high-density area is observed directly below the nipple, but the shape is flat, the internal density is homogenous, and there are no signs of invasiveness around it, making it difficult to diagnose a recurrence based on this image alone.

**Fig. 2:**
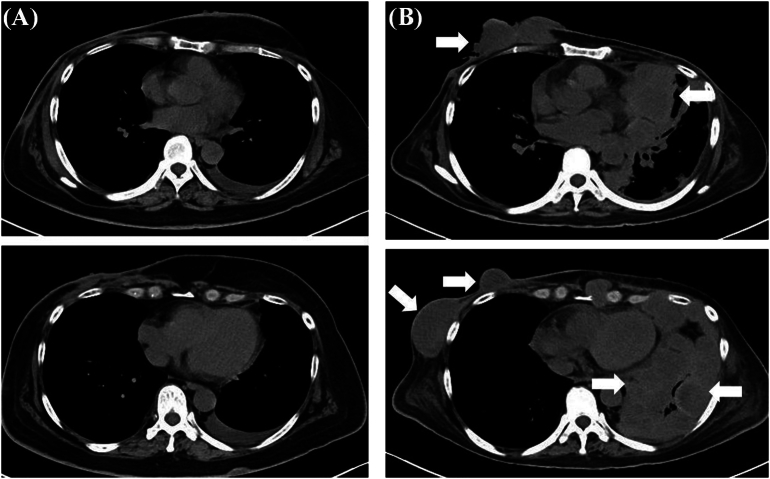
Simple Thoracic CT; (A) 4 weeks and (B) 17 weeks after operation of right local recurrence. The right chest wall mass and left pleural tumor grew rapidly, and after 17 weeks, the left thoracic cavity was almost 70 % occupied by a pleural mass and the right chest wall mass grew to 10 cm in size.

**Fig. 3:**
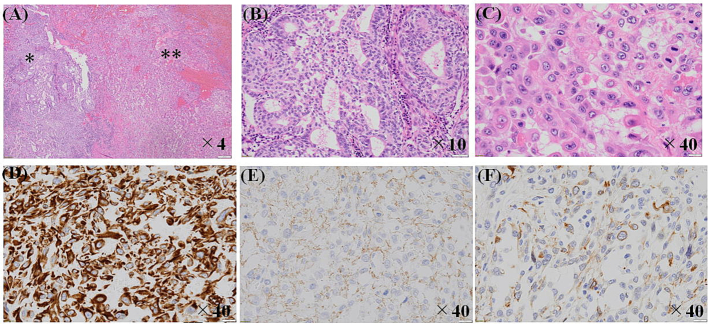
Pathological findings: (A) pleomorphic carcinoma (**) transitioning from invasive ductal carcinoma (*) (HE ×4). (B) Typical invasive ductal carcinoma (* in A) (HE ×10). (C) Pleomorphic carcinoma (** in A) (HE ×40). (D) is clear positive expression of Vimentin (×40), (E) is faint positive expression of E-cadherin (×40), and (F) is weak positive expression of AE1/3 (×40).

A 49-year-old woman was diagnosed with bilateral breast cancer (Rt: cT2cN0M0 cstageIIA, Lt: cT2cN1M0 cstageIIB) and its intrinsic type was non-luminal type (Estrogen & Progesterone receptor positive, human epidermal growth factor receptor 2 (HER2) 3+, ki67 31 %) in the right side and lumina A-like (Estrogen & Progesterone & HER2 negative, ki67 < 5 %) in the left side. As neoadjuvant chemotherapy, 4 cycles of adriamycin and cyclophosphamide (60/600 mg/m^2^) every three weeks, followed by paclitaxel (80 mg/m^2^) and trastuzumab (loading 4 mg/kg, maintenance 2 mg/kg) given weekly for 12 weeks were administered, and the patient achieved clinical partial response on imaging. Bilateral breast-conserving surgery and axillary lymph node dissection were performed, and the pathological evaluation of therapeutic effect was Grade 1 (Rt: ypT1bypN0M0 ypstageI, Lt: ypT1cypN0(i+)M0 ypstageI). After radiotherapy (50Gy/25Fr) of the residual mammary gland, trastuzumab (6 mg/kg) was administered but the patient requested to discontinue treatment after 2 courses. Thereafter, the patient was subsequently followed with endocrine therapy (Tamoxifen). 6 years and 7 months after the initial operation, the patient visited the hospital complaining of swelling and erosion of the right nipple. Computed Tomography Scan (CT) showed a swollen right nipple (Fig. 1), and 3 stump cytology tests for the erosion were negative, but the nipple itself showed a tendency to grow, so a core needle biopsy (CNB) was performed. The pathological findings showed invasive carcinoma, and suggested metaplastic carcinoma. As no metastases to other organs were found, a right mastectomy was performed. After chemotherapy using carboplatin, radiation therapy to the supraclavicular fossa was planned, but 4 weeks after the operation, a round mass of approximately 1 cm was found in the B area of the left breast. CNB revealed a suspicious malignant metaplastic carcinoma. It was determined that there was a high possibility of metastasis to the contralateral side, a left mastectomy was performed. However, 12 weeks later, a left pleural tumor and a right chest wall mass appeared, and after a further 13 weeks, the left thoracic cavity was occupied by almost 70 % by a pleural mass, and the right chest wall mass grew to 10 cm in size (Fig. 2). Furthermore, the WBC count rose rapidly to 98.8 × 103/μL (Ne 94.8, Lym 2.5, Mono 1.9). Bone marrow metastasis was suspected but bone marrow aspiration could not be performed due to the patient's poor general condition. The patient passed away 25 weeks later after the diagnosis of local recurrence. Pathological examination showed PC transitioning from ductal carcinoma, and the subtype was triple negative breast cancer (TNBC). Based on immunostaining, AE1/3 (Anti-pan Cytokeratin), Beta-catenin, E-cadherin and Vimentin were positive, and CAM5.2 (cytokeratin), HBM45 (human melanin black45) and LCA (Leukocyte Common Antigen) were negative. It was a highly malignant transformation of ductal carcinoma, and it took the form of a so-called dedifferentiated carcinoma, consisting of differentiated and undifferentiated components (Fig. 3).

## 3 Discussion

PC of the breast is extremely rare, accounting for <0.1 % of all breast malignancies, and it apparently occurs most frequently during the perimenopausal period [4]. It is highly malignant, and Silver et al. [2] point out that the most common characteristic is rapid growth, with palpable masses appearing in 65 % of cases over a period of several weeks to two years. Invasion of the skin and pectoral muscles is not uncommon, and the disease progresses quickly to distant and lymph node metastases [4]. In line with these previous reports, our case also had an astonishing rate of progression.

Due to the small number of cases of PC, imaging tests have hardly been studied. PC often does not show typical cancer findings, and it has been reported that mammography and ultrasound are not useful [2]. However, it has also been reported that in cases of large tumors (average tumor diameter 7.8 cm), the tumors tend to show a cystic mass appearance due to cavitation caused by central necrosis [6]. In our case, the right nipple was enlarged compared to the opposite side, but there was no clear evidence of marginal irregularity, making it difficult to diagnose cancer through imaging tests.

PC is usually diagnosed by pathological examination, but the pathological findings via CNB in our case suggested metaplastic carcinoma, and a diagnosis of PC could not be made. Silver et al. [2] reported that spindle cell infiltration was observed in 31 % of PC cases, Nguyen et al. [3] reported 38 %, and Jing et al. [4] reported 20 %. In diagnosing PC, it is important to differentiate it from other malignant tumors such as giant cell or spindle cell sarcomas. Silver et al. [2] reported that 35 % of PC cases were diagnosed as sarcomas or possible sarcomas. In particular, in cases where the tumor border is clearly fine, there have been reports of misdiagnosis as metastatic tumors [4]. In terms of pathological diagnosis of PC, it is important to carefully search for areas where PC shows transition from invasive ductal carcinoma. It is also important to distinguish between PC and metaplastic carcinoma. For example, it is essential that there are no heterologous components including bone, cartilage, and striated muscle as non-epithelial tissues of the tumor for a definite diagnosis of PC. When both epithelial and heterologous components look malignant, carcinosarcoma should be considered. Immunostaining can serve as a substantial tool for making a diagnosis of histological variants represented by PC. Specifically, immunohistochemical expression of epithelial markers such as a cytokeratin may be helpful for the diagnosis of PC. For example, PC can be differentiated from pleomorphic lobular carcinoma with positive reaction of e-cadherin. Jing et al. [4] reported that bizzare giant cells are also observed in sarcomas, but that immunostaining for epithelial and mesenchymal markers is useful for diagnosis because epithelial morphology of other tumor components is usually missing in sarcomas. In addition, it has been reported that the following points are helpful in distinguishing cancer from the osteoclastic giant cells: osteoclastic giant cells and fibroblasts are negative for epithelial markers [7], and osteoclastic giant cells are often regularly shaped, have round to ovoid nuclei, and are not hyperchromatic [3]. There are multiple reports showing that TNBC is the most common subtype of PC [[2], [3], [4],^8^], but one study showed that HER2 is overexpressed [1].

Very little research has been conducted on postoperative treatment for PC, which may partly be explained by the fact that it is rare but this also could be related to the unique pathological features of this disease, where the rate of progression is so rapid that treatment cannot keep up, as in our case. Treatment often consists of surgery [2,9,10], and the commonly chosen procedure is mastectomy, given that the tumor tends to be large at the time of diagnosis, and have high-grade characteristics, with increased likelihood that lymph node metastasis exists [9]. Regarding chemotherapy, there has been one reported case in which cyclophosphamide, epirubicin, and fluorouracil were administered as an adjuvant chemotherapy after operation, and there was no subsequent recurrence [11]. However, there are no reports of chemotherapy that is effective for PC, and existing anticancer drugs has been ineffective [12]. Furthermore, there are reports of cases in which recurrent disease was resistant to chemotherapy and/or radiation therapy [13].

Based on previous studies, the prognosis of PC is known to be poor [[2], [3], [4]], Nguyen et al. [3] reported that a tumor size of 5 cm or more and the presence of spindle cell infiltration were independent poor prognostic factors, and that the 5-year survival rates differed significantly between those with and without spindle cell infiltration (38 % and 89 %), and between tumor size ≥5 cm and <5 cm (39 % and 87 %). The recurrence rate is very high, and metastases occur in a wide variety of locations, including the liver, lungs, pleural surfaces, vertebral bone, and local recurrence [2]. According to Silver et al. [2], of 16 patients with follow-up, 6 (38 %) were disease-free (mean, 74 months), 4 (25 %) alive with disease (mean, 33 months) and 6 (38 %) died at a mean of 22 months. In our case, local recurrence occurred despite negative surgical margins, suggesting that the cancer spread widely via blood vessels and lymphatic vessels. This is also thought to be a factor that contributes to the high recurrence rate.

## 4 Conclusion

When a rapidly growing breast mass is encountered, it is necessary to consider PC as one of diagnostic candidate. Little research has been done on PC, particularly on diagnosis and therapy. Thus, further case accumulation and analyses are awaited to gain more insight on the characteristics of PC.

## Author contribution

NT (Nobuyuki Takemoto) performed the operation, chemotherapy, and outpatient follow up of the patient, conducted the literature review, and wrote the manuscript. MY (Masanori Yasuda) performed the pathological examination. KS (Kousuke Shimanaka) and HY (Hiroshi Yamamoto) assisted in the operation. All authors read and approved the final manuscript.

## Consent

Written informed consent was obtained from the patient and family for publication of this case report and accompanying images. A copy of the written consent is available for review by the Editor-in-Chief of this journal on request.

## Ethical approval

Our institution, Japan Medical Alliance East Saitama General Hospital, does not require ethical approval for case reports that are deidentified and collected retrospectively.

## Guarantor

Nobuyuki Takemoto.

## Research registration number

This is a case report, and no database approval was applied.

## Funding

This research did not receive any specific grant from funding agencies in the public, commercial, or not-for-profit sectors.

## Conflict of interest statement

The authors declare that they have no conflict of interest.

## Figures and Tables

**Fig. A:**
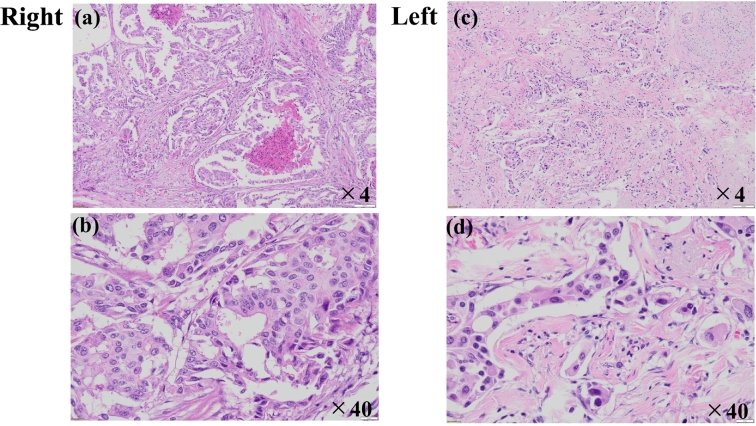
Pathological findings of the bilateral primary breast cancers: invasive ductal carcinoma after NAC (HE). (a), (b) (right): The tumor of invasive ductal carcinoma (NG1), measuring 0.9 × 0.8 cm, with a stage at pT1b and Grade 1a therapeutic effect, but the ductal carcinoma in situ (DCIS) component spread to the surrounding the area, measuring 5.5 × 5 cm. Neither lymphatic nor vascular invasion was noted (Ly0, V0). The surgical margin was negative. No reginal lymph node metastasis was noted (pN0: 0/4). (c), (d) (left): The tumor of invasive ductal carcinoma (NG2) spread over the area from the center of the specimen, measuring 3.5 × 2.5 cm, with a stage at pT1c and Grade 1b therapeutic effect. Lymphatic and vascular invasion was noted (Ly1, V1). The surgical margin was negative. Isolated lymph node metastasis was noted (pN0(1/6, (i+))).

**Fig. B:**
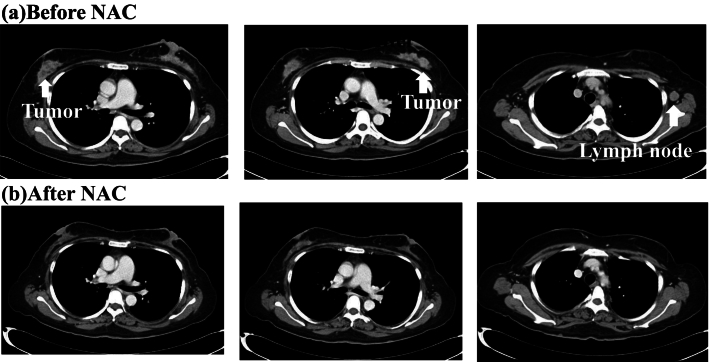
Enhanced CT images before and after NAC. (a) Before NAC: The breast masses measuring approximately 30 mm in size at the right 12 o'clock position and approximately 21 mm in size at the left 3 o'clock position were found, as well as a lymph node metastasis measuring approximately 10 mm in size in the left axilla. No metastasis was found in the lungs, liver, or spine. (b) After NAC: The bilateral breast tumors and left axillary lymph node disappeared, and CT scan showed clinical complete response. No new distant metastases were observed.

**Fig. C:**
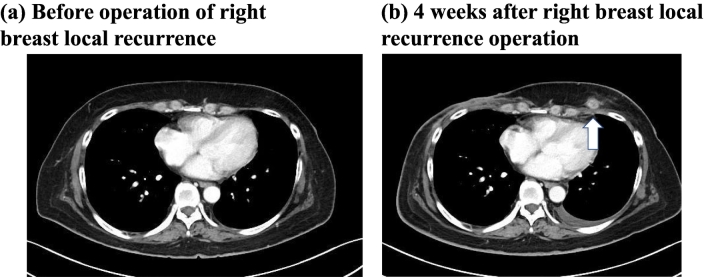
Metastasis to lt. mammary gland. 4 weeks after the operation, a tumor of approximately 1 cm in size with an unclear border and a partially spiculated appearance was detected in the left B area. A core needle biopsy was performed, and the diagnosis was suspected metaplastic carcinoma.

**Fig. D:**
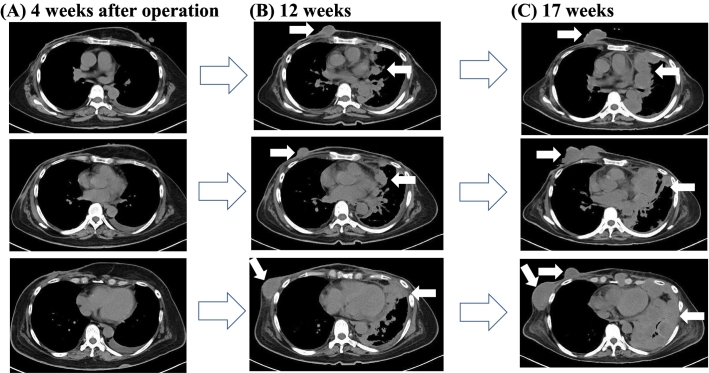
Rapidly growing recurrent tumor in the right chest wall and left thoracic cavity. Thoracic CT Scan (A) 4 weeks, (B) 12 weeks and (C) 17 weeks after operation of right local recurrence. The right chest wall mass and left pleural tumor grew rapidly, and after 17 weeks, the left thoracic cavity was almost 70 % occupied by a pleural mass and the right chest wall mass grew to 10 cm in size.

**Fig. E:**
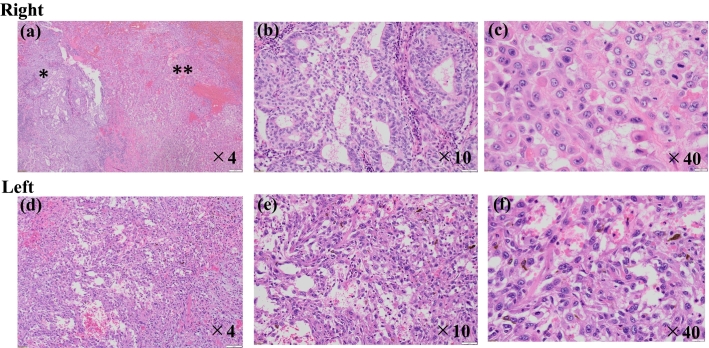
Pathological findings of the bilateral breast cancers: pleomorphic carcinoma (PC) showing recurrence (right) and metastasis (left) (HE). (a), (b), (c) (right): Invasive ductal carcinoma (*) and PC (**) showed a transition between both (a) (b: invasive ductal carcinoma, c: PC). (d), (e), (f) (left): The metastasis of the right recurrent tumor showed PC alone.
